# Is recent Afghanistan survey data suitable for fertility analysis? A regional investigation based on fertility inhibiting determinants

**DOI:** 10.1371/journal.pone.0223111

**Published:** 2019-10-16

**Authors:** Jamal Abdul Nasir, Sohail Akhtar, Syed Arif Ahmed Zaidi, Andleeb Rani, Hina Bano, Andrew Hinde

**Affiliations:** 1 Department of Statistics, Government College University, Lahore, Pakistan; 2 Department of Community Medicine, Quaid-e-Azam Medical College, Bahawalpur, Pakistan; 3 National College of Business Administration & Economics, Rahim Yar Khan, Pakistan; 4 Department of Social Statistics and Demography, University of Southampton, Southampton, United Kingdom; Peking University Third Hospital, CHINA

## Abstract

Afghanistan has been a country blighted by war over the past five decades and limited research is available on its demography. This study seeks to assess the suitability of recent survey data for Afghanistan (the 2010 Afghanistan Mortality Survey (AMS)and the 2015 Afghanistan Demographic and Health Survey (ADHS)) for estimating levels and trends in fertility. As several fertility measures rely on the quality of age data, we first apply demographic tools for the identification of age misreporting, finding evidence that it is severe. We then explore the consistency of fertility reporting across the two surveys, finding that the 2015 ADHS reports higher fertility among older women than the 2010 AMS although the seasonal pattern of fertility is consistent across the two surveys. We then estimate total fertility rates in 2008–2010 and 2012–2015 and measures of Bongaarts’s key proximate determinants of fertility for Afghanistan and its provinces for urban and rural areas separately. The results show that fertility is similar in urban and rural Afghanistan. Although most of the provincial data on the proximate determinants is reasonably consistent with the fertility rates, there are anomalies in some provinces which indicate the possible under-reporting of births. Overall, we conclude that the fertility data in the two surveys can be used with care to give an indication of broad regional fertility patterns and trends in the country.

## Introduction

Many aspects of the demography of the Islamic Republic of Afghanistan remain under-researched. The exact population of the country is not known due to the lack of a recent census. According to the Central Statistics Organization of Afghanistan (CSOA) the total population of the country is estimated to be 32.2 million people [1]. The urban population (24 per cent) is only one third of the rural population (71 percent). Afghanistan’s population and socio-economic development has faced the challenge of war over the past five decades, and unrest and political instability still affect the living conditions and fertility choices of inhabitants. According to the Population Reference Bureau, among the countries of the South Asian region, Afghanistan has the highest total fertility rate (an estimated 4.8 children per woman in 2018) [2].

The fertility transition in Afghanistan has attracted limited attention in demographic research [3]. Although there have been several surveys co-ordinated by the CSOA, including the Afghanistan Multiple Indicator Cluster Surveys (1997, 2000, 2003, 2010–2011), Afghanistan Living Conditions Survey (2005, 2007–2008, 2011–2012, 2013–2014, 2016–2017), a Socio-Demographic and Economic Survey (2016) at provincial level (the province is the first level administrative unit of the country), and the Afghanistan Health Survey (2003, 2005, 2007–2008, 2015), these surveys did not collect birth history data. In addition, most surveys coordinated by the CSOA are not widely available to researchers outside the country.

Neighboring countries of the South Asian region have shown a noticeable decline in fertility. For instance, Iran’s total fertility rate is 2.0 and that of Pakistan is 3.1 [2]. Contraceptive prevalence in Iran among married women of reproductive age is 77 per cent, compared with 35 per cent in Pakistan and only 23 per cent in Afghanistan (for modern methods, the corresponding percentages are 57, 26 and 20) [2]. Contraceptive use is one of the three major proximate determinants of fertility as proposed by John Bongaarts [4,5].

In this paper we examine the quality of the data on fertility collected in the only two recent surveys that did include birth histories: the Afghanistan Mortality Survey carried out between April and December 2010 (hereafter 2010 AMS) and the Afghanistan Demographic and Health Survey conducted between June 2015 and February 2016 (hereafter 2015 ADHS).

There exists an enormous literature on the types of error (examples include omission, duplication, misreporting, telescoping due to memory lapse) affecting estimates of fertility [6,7]. In this paper we focus on the extent of age misreporting before examining the consistency of fertility estimates from the two surveys. By comparing two surveys conducted only five years apart, we hope to be able to identify where the fertility outcomes are inconsistent and therefore indicate errors or omissions in at least one of the surveys, and also to emphasise topics where the surveys produce consistent results, which increases our confidence that the data are accurate. We then estimate total fertility rates in 2008–2010 and 2012–2015 and measures of Bongaarts’s key proximate determinants of fertility for Afghanistan and its provinces for urban and rural areas separately.

## Data

The 2010 AMS used a sampling frame provided by the CSOA. It aimed to provide estimates of demographic variables which were representative of the whole country, and of urban and rural areas within three domains [8]. The domains were (1) North (the Northern and North Eastern regions), (2) Central (the Western, central highland and Capital regions), and (3) South (the Southern, South Eastern and Eastern regions). A two-stage sampling process was used. The country was divided into strata based on urban and rural residence and the three domains listed above (additional strata were used for rural areas within each of the domains). Within each stratum enumeration areas were selected in a first stage, and then households were selected within each enumeration area in a second stage. Interviews were conducted with all women aged 12–49 years in the selected households. In all, 23,897 households were selected, and 22,381 were successfully contacted [8]. A total of 47,848 women were interviewed. A small number of areas of the country were not covered in the survey for security reasons [8]. The population sampled represents 87 per cent of the total population, and most of the omitted areas were in the Southern region [8]. The urban areas of Kabul were heavily over-sampled.

The 2015 ADHS used an updated version of a sampling frame provided by the CSOA. The sampling frame used information about 34 provinces, control areas, districts and urban or rural residence. In the 2015 ADHS a two-stage stratified sample design was used [9]. The first stage involved selecting 950 clusters (260 in urban areas and 690 in rural areas). Due to security issues in some areas of Afghanistan 101 reserve clusters were preselected, giving a total of 1,051 clusters for the survey. Of these, 75 clusters were classified as insecure during the household listing operation; for the 976 remaining clusters household listing was successfully completed. Eventually the survey was carried out in 956 clusters. The second stage used systematic random sampling of households. A total of 25,741 households were selected for the sample, and from these households 30,434 ever-married women age 15–49 years were identified for individual interviews and 29,461 interviews were successfully completed [9]. Some areas, notably Nooristan province, were over-sampled,

The 2015 ADHS only interviewed ever-married women aged 15–49 years. For comparative purposes, we compare these women with the 26,730 ever-married women in the same age range in the 2010 AMS. The distribution of these women in both surveys by province and urban-rural residence is presented in Table 1. Although the sampling fractions varied by province and from survey to survey, both surveys provided weights, by applying which they may be rendered representative of the national population. When comparing national estimates, therefore, we use weighted data.

**Table 1:** Number of ever-married women aged 15–49 years by province and urban/rural residence: Afghanistan Mortality Survey 2010 and Afghanistan Demographic and Health Survey 2015.

Region	Province	2010 Afghanistan Mortality Survey (unweighted)		2015 Afghanistan Demographic and Health Survey (unweighted)	
		Urban	Rural	Urban	Rural
Northern	Balkh	746	811	325	584
Northern	Faryab	192	745	274	468
Northern	Jawzjan	243	414	308	557
Northern	Samangan	130	306	206	476
Northern	Sar-E-Pul	72	547	192	620
North Eastern	Badakhshan	46	1,000	226	609
North Eastern	Takhar	210	902	275	544
North Eastern	Baghlan	371	774	294	446
North Eastern	Kunduz	384	647	331	508
Western	Badghis	33	292	159	716
Western	Farah	35	468	168	965
Western	Ghor	34	449	170	716
Western	Herat	622	1,074	316	673
Central Highland	Bamyan	0	246	154	498
Central Highland	Daykundi	0	260	110	559
Capital	Kabul	2,799	385	458	297
Capital	Kapisa	0	239	24	850
Capital	Logar	38	261	89	826
Capital	Panjsher	0	86	0	681
Capital	Parwan	199	311	211	533
Capital	Wardak	0	354	67	803
Southern	Kandahar	634	0	430	522
Southern	Helmand	230	0	283	560
Southern	Nimroz	76	144	279	401
Southern	Ghazni	95	1,283	174	972
Southern	Urozgan	47	318	162	643
Southern	Zabul	47	0	172	0
South Eastern	Khost	52	1,156	315	1,023
South Eastern	Paktika	0	507	0	1,110
South Eastern	Paktya	111	1,000	237	937
Eastern	Kunarha	40	861	209	525
Eastern	Laghman	0	605	147	653
Eastern	Nangarhar	490	2,032	260	763
Eastern	Nooristan	0	277	0	1,398
	Total	7,976	18,754	7,025	22,436

Sources: Afghanistan Mortality Survey 2010 [8]; Afghanistan Demographic and Health Survey 2015 [9].

Note: There are no urban areas in Nooristan and Panjsher provinces. In 2015, rural areas of Zabul province could not be sampled because of security issues. In 2010 rural areas of Zabul, Helmand and Kandahar provinces could not be sampled because of security issues. The sampling frame for the 2010 survey divided the country into three domains: North, Central and South, and sampled urban households within each domain without further stratification. Hence the number of households in the sample in some provinces with small urban populations (Kapisa, Wardak, Laghman, Daykundi and Paktika) was zero. It is not clear why no urban households were sampled in Paktika province in 2015. In the 2010 survey we have excluded women aged 12–14 years and never-married women, so that we have a group that is directly comparable to the sample in the 2015 survey.

## Methods

This paper examines the quality of the birth history data in the two surveys using the following approaches. First, we measure age misreporting using two well-known methods: Whipple’s Index and Myers’s Blended Index. Whipple’s Index measures age heaping on ages with digits ending in 0 and 5 in the adult age range [10]. Its normal application uses the age range 23–62 years and the formula

$$\mathrm{Whipple}\;\mathrm{Index}=\frac{P_{25}+P_{30}+P_{35}+P_{40}+P_{45}+P_{50}+P_{55}^{}+P_{60}}{\left(\frac{1}{5}\right)\sum_{x=23}^{x=62}P_{x}}x\;100.$$

Because we only have data on women aged 15–49 years, we calculate Whipple’s Index using the age range 18–47 years.

Modifications and extensions to Whipple’s Index have been suggested in the literature [11,12]. Here, however, we use the original version designed to measure heaping on ages ending in the digits 0 and 5. This is the dominant form of age heaping in Afghanistan. Myers’s Blended Index is a more general measure, which takes into account preferences for (or antipathy towards) ages ending in any digit [10,13].

Second, we look at the consistency of the estimates of fertility produced by the birth histories in the two surveys. We do this in two ways, first by looking at the seasonal pattern of births and, second, by computing age-specific fertility rates (ASFRs) and the total fertility rate for four-year periods from 1984–1987 to 2012–2015 using data from the two surveys. Because both surveys are designed to be representative of the majority of the population, ASFRs for the whole country estimated for the same four-year period using weighted data should be similar for both surveys. We compute the ASFRS using weighted data to maximise comparability. The ASFRs are computed using the exact exposure method. The numerators are the total numbers of births reported by the women in each survey in each four-year period when they were in each five-year age group at the time of giving birth. The denominators are obtained by working out the exact exposure (in years and fractions of a year) for each woman in each age group in each four-year period and summing these over all women.

Third, we use the ASFRs in the most recent period for each survey to estimate total fertility rates for the urban and rural populations in each province. To maximise comparability, we have amalgamated strata with fewer than about 200 women in either survey to produce a set of sub-samples defined on the basis of province and urban-rural residence which are comparable across the two surveys and which include approximately 200 women or more. We estimate the total fertility rate for each of these sub-samples for the period 2008–2010 from the 2010 AMS, and for the period 2012–2015 from the 2015 ADHS.

We then examine some key determinants of fertility in the same set of sub-samples. John Bongaarts, in his classic work, built on the ideas of Kingsley Davis and Judith Blake, and identified the four most important proximate determinants of the fertility outcome in any population: the prevalence of marriage, the use of contraception, the use of abortion and the impact of breastfeeding [4,5,14]. Here, we focus on marriage, contraception and breastfeeding. For each of the provincial and urban/rural sub-samples we compute a measure of the likely impact of each of these determinants on fertility. For marriage, we calculate the percentage of 20–24 year old women who are currently married, using the entire sample from 2010 AMS (which interviewed both ever-married and never married women). For contraception, we compute the percentage of ever-married women aged 15–49 years who are using a modern contraceptive method at the time of the 2015 ADHS. Finally, for breastfeeding we measure the percentage of the most recent births to women in the 2015 ADHS born 12–23 months before the survey who were still being breast fed on the survey date. We expect high fertility to be associated with a high proportion of 20–24 year olds being married, a low prevalence of modern contraception and a low proportion of babies aged 12–23 months being breast fed.

## Results

**Fig 1:**
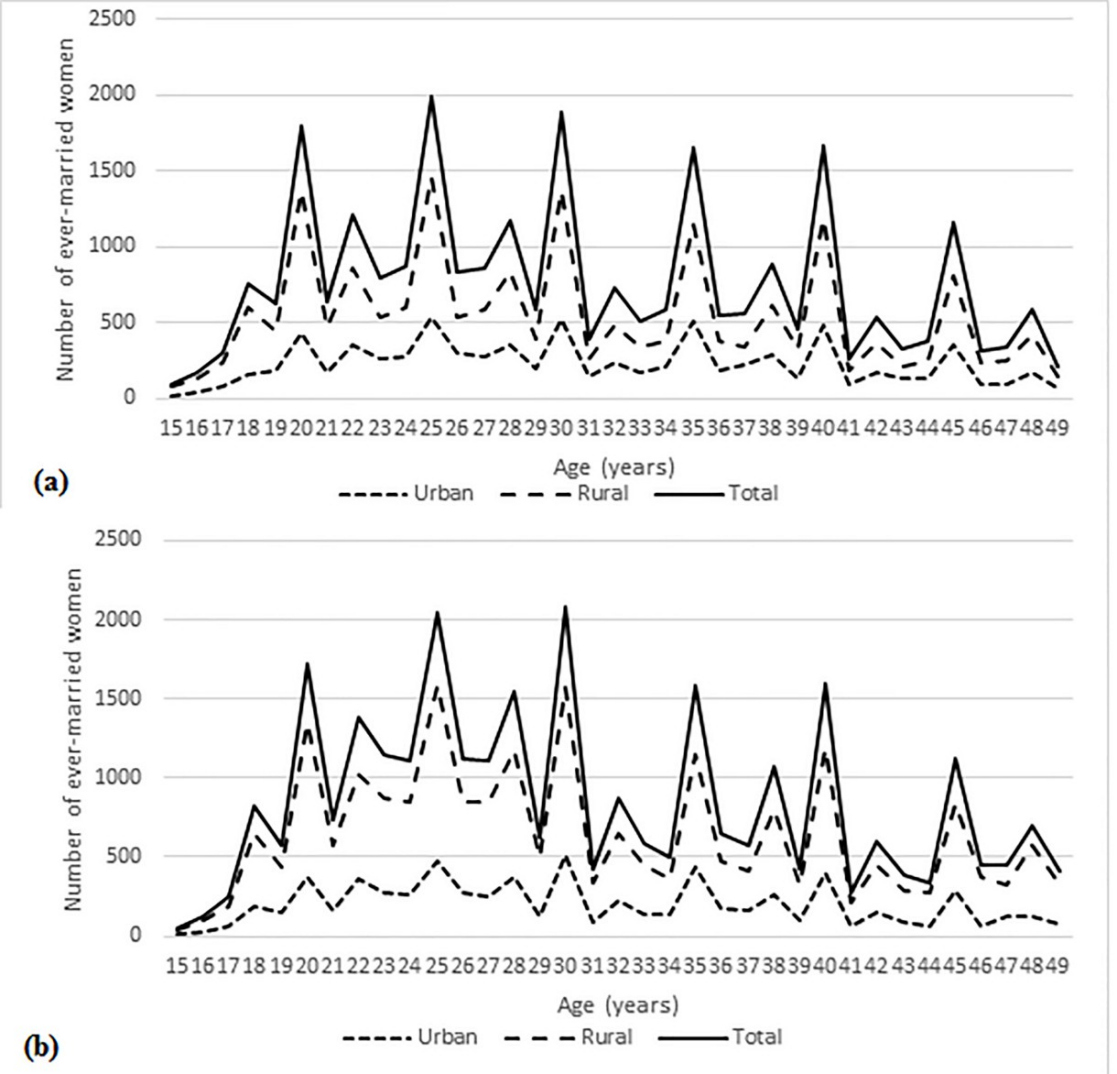
**Age distribution for ever-married women aged 15–49 years: (a) Afghanistan Mortality Survey 2010 and (b) Afghanistan Demographic and Health Survey 2015.** Sources: Afghanistan Mortality Survey 2010; Afghanistan Demographic and Health Survey 2015. Note: These figures use unweighted data.

Fig 1 presents the distribution by single years of age of the samples from the two surveys, distinguishing women living in urban and rural areas. The age distribution is highly irregular, with clear peaks on ages ending in the digits 0 and 5 (and to a lesser extent 2 and 8). This indicates substantial age misreporting by respondents. The degree of age misreporting is not obviously different in the two surveys, neither is it clearly greater in rural than in urban areas.

Table 2 presents the values of two indices of age misreporting at the national and provincial level in Afghanistan for urban and rural samples in 2010 and 2015. Whipple’s Index measures age heaping on ages with digits ending in 0 and 5 [10]. According to United Nations criteria, values of the index above 175 indicate ‘very rough’ age reporting and values between 125 and 175 indicate ‘rough’ age reporting. It is clear that in almost all of Afghanistan’s provinces age reporting is ‘rough’, and in most it is ‘very rough’. The highest levels of age misreporting occur in the Central Highlands region and, in 2010, in Eastern region.

**Table 2:** Age misreporting indices by province and urban-rural residence: Afghanistan 2010 and 2015.

Region	Province	Whipple’s Index				Myers’s Blended Index			
		2010 Afghanistan Mortality Survey		2015 Afghanistan Demographic and Health Survey		2010 Afghanistan Mortality Survey		2015 Afghanistan Demographic and Health Survey	
		Urban	Rural	Urban	Rural	Urban	Rural	Urban	Rural
Northern	Balkh	158	215	178	153	13.5	23.2	18.3	13.3
Northern	Faryab	201	209	171	121	21.6	23.7	17.3	12.3
Northern	Jawzjan	189	183	193	148	22.7	21.2	24.6	18.6
Northern	Samangan	216	218	205	198	26.4	23.3	25.0	27.9
Northern	Sar-E-Pul		222	197	200		27.9	23.8	21.7
North Eastern	Badakhshan	223	226	186	214	24.8	27.7	28.3	28.1
North Eastern	Takhar		230	164	209		29.5	16.5	23.6
North Eastern	Baghlan	205	232	159	165	27.0	29.2	12.7	18.0
North Eastern	Kunduz	207	195	178	173	21.7	23.4	14.7	15.5
Western	Badghis	175	189	203	259	16.5	16.7	25.7	39.3
Western	Farah		160		149		18.9		14.3
Western	Ghor		242		178		26.3		21.0
Western	Herat		206	160	156		23.8	19.2	18.8
Cen. Highland	Bamyan		277	240	262		36.5	31.2	34.0
Cen. Highland	Daykundi		246		267		27.6		39.5
Capital	Kabul	187	215	166	225	19.6	26.7	18.1	31.3
Capital	Kapisa	202	234	177	167	21.8	29.9	20.5	16.9
Capital	Logar		141		150		19.4		15.0
Capital	Panjsher		219		227		25.8		29.0
Capital	Parwan				174				17.1
Capital	Wardak		174		205		19.4		23.1
Southern	Kandahar	149	na	188	183	13.6	na	26.8	24.4
Southern	Helmand	119	na	182	171	15.7	na	19.6	14.4
Southern	Nimroz		175	217	208		13.5	27.3	27.1
Southern	Ghazni	199	156	165	148	29.2	19.5	17.1	13.9
Southern	Urozgan		181		136		16.9		13.7
Southern	Zabul		na		na		na		na
South Eastern	Khost	227	175	215	185	32.7	19.2	27.3	24.7
South Eastern	Paktika		181		150		23.0	na	11.8
South Eastern	Paktya		206	172	162		24.9	28.2	16.6
Eastern	Kunarha	228	236	197	214	31.0	32.8	29.1	28.9
Eastern	Laghman		115		203		19.1		26.9
Eastern	Nangarhar		244	165	186		33.9	21.2	22.6
Eastern	Nooristan	na	254	na	161	na	35.4	21.1	14.9
Whole country		186	207	183	181	19.5	24.0	21.1	20.0

Sources: Afghanistan Mortality Survey 2010 [8]; Afghanistan Demographic and Health Survey 2015 [9]. Adjacent provinces within regions have been combined where samples were small.

Note: This table uses unweighted data. Whipple’s Index measures the extent of heaping on ages with digits ending on 0 and 5. It expresses the number of women reporting ages ending in digits 0 and 5 as a percentage of the number to be expected if ages were correctly reported. It is here calculated using women aged 18–47 years. Myers’s Blended Index is a summary measure of preferences for or tendencies to avoid ages ending in all digits [13]. Its value ranges from 0 (no heaping) to 90 (everyone reports an age ending in the same digit). na–not available.

The pattern revealed by Myers’s Blended Index is similar to that for Whipple’s Index; in particular, in no province is the value of Myers’s Blended Index below 10, indicating substantial levels of preference for certain terminal digits in all areas of the country.

An important feature of age reporting in Afghanistan is that it is not obviously better in urban areas than in rural areas in either 2010 or 2015 (Table 2). It is, however, slightly better among women with some education than among women with no education. In 2010, Whipple’s Index was 158 for women with some education compared with 207 for women with no education; the corresponding values in 2015 were 155 and 186. Myers’s Blended Index took the values 14.3 and 23.8 among women with some education and no education respectively in 2010; by 2015 the corresponding values were 13.1 and 21.3. Taken together, these results suggest a slight improvement in age reporting in rural areas between 2010 and 2015, but very little improvement in urban areas.

**Fig 2:**
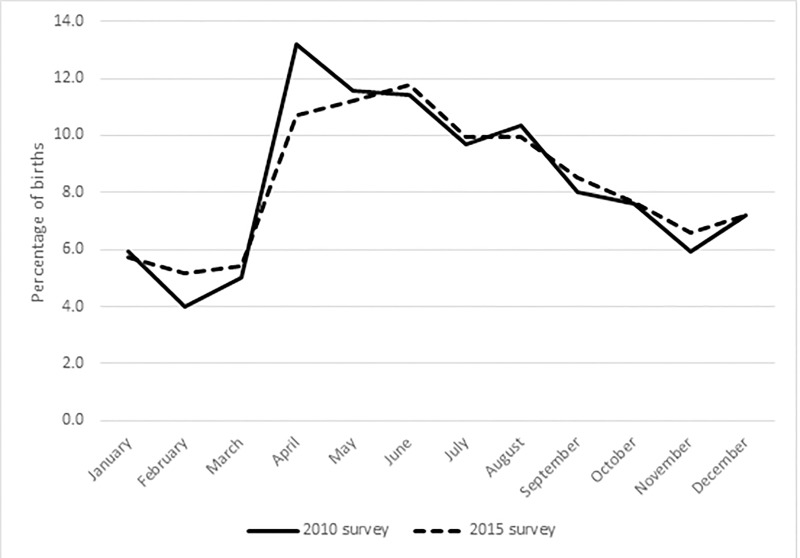
Percentage of births in each month in Afghanistan: Afghanistan Mortality Survey 2010 and Afghanistan Demographic and Health Survey 2015. Sources: Afghanistan Mortality Survey 2010; Afghanistan Demographic and Health Survey 2015.

We turn now to the comparison of fertility patterns revealed by the birth history data in the two surveys. Fig 2 shows the percentage of births in each calendar month according to the 2010 AMS and the 2015 ADHS. The pattern is very similar in the two surveys. There is a pronounced seasonal pattern, with births being fewest in February and March, and most in April, May and June. This implies that conceptions reach a low point in May and June, and a peak in July, August and September. the difference in the number of births between the trough in February and March and the peak in April, May and June, is very substantial, and among the highest reported from national populations [15]. The peak is not an artefact of the imputation of the month of birth for some of the births: the proportion of births for which imputation was carried out was much too small in either survey to account for anything more than a tiny fraction of the seasonal pattern.

**Fig 3:**
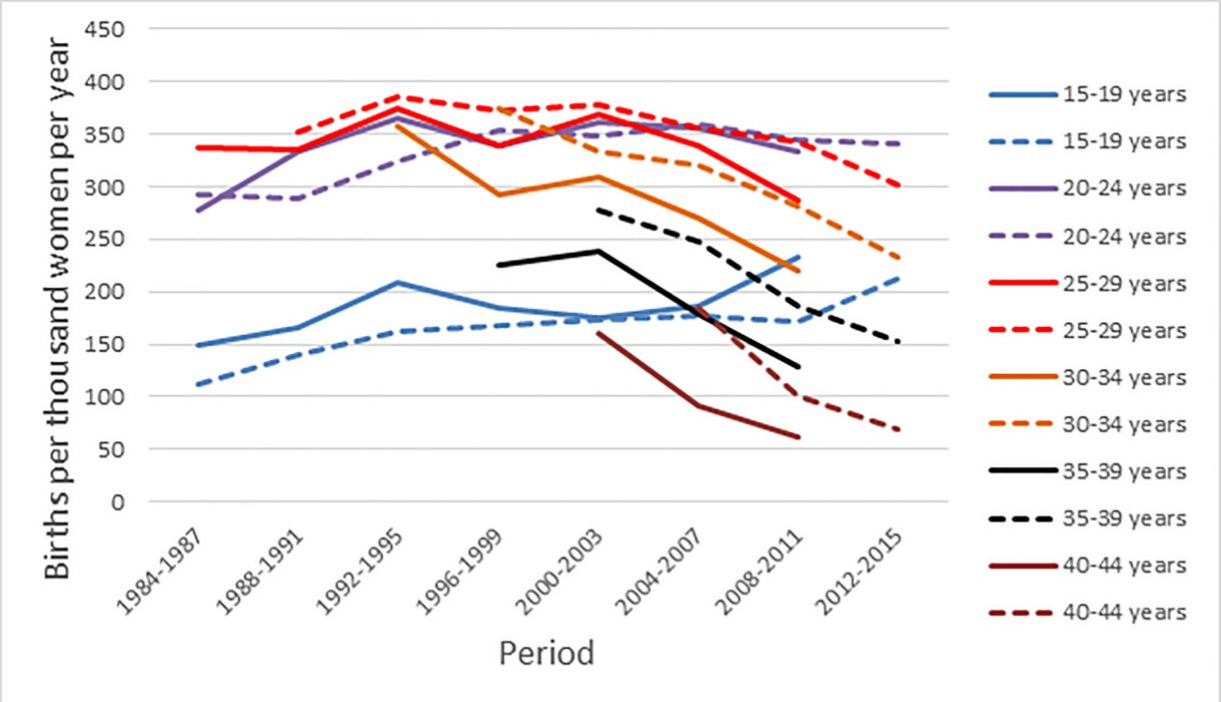
Age-specific fertility in five-year age groups by four-year periods: Afghanistan Mortality Survey 2010 and Afghanistan Demographic and Health Survey 2015. Sources: Afghanistan Mortality Survey 2010; Afghanistan Demographic and Health Survey 2015.

Fig 3 shows the age-specific fertility rates (ASFRs) revealed by the birth history data for the six five-year age groups from 15–19 to 40–44 years in four-year periods from 1984–1987 to 2012–2015. There is evidence of a decline in fertility at ages 25–29 years and over since around 2000, suggesting that Afghanistan has entered the fertility transition as first observed by Thomas Spoorenberg using the 2010 AMS [3]. However, a second pattern in Fig 3 is that the 2015 ADHS generates lower ASFRs than does the 2010 AMS for the age group 15–19 years and higher ASFRs in all periods than does the 2010 AMS for age groups 25–29 years and older. The difference is substantial for age groups 35–39 and 40–44 years. The lower reported fertility at older ages in the 2010 AMS merits further investigation. One possible reason, a lower proportion of older women currently married in the 2010 AMS, can be ruled out, as typically more than 90 per cent of women were currently married at all ages up to 49 years in both surveys. Another reason is a tendency to under-report female births, which was noted in the report to the 2010 AMS [8]. There is some evidence that this tendency was greater among older women in 2010 AMS than in 2015 ADHS. The sex ratio of births to women aged 40–44 years was 115 boys per 100 girls in the 2010 AMS and 110 boys per 100 girls in the 2015 ADHS. Finally, some of the difference is the result of the different distribution of women by age *within* each the age groups arising from the fact that we are estimating period fertility using data from a cohort aged 15–49 years at the date of interview.

The total fertility rate for the period 2008–2011 based on the 2010 AMS was 6.46 and that for the same period based on the 2015 ADHS was 7.05. This also represents a substantial difference. The total fertility rate for the period 2012–2015 based on the 2015 ADHS is 6.61, still higher than that reported for the earlier period by the 2010 AMS, but marking a decline in fertility since 2008–2011.

The ASFRs reported in Fig 3 relate to ever-married women. As such they cannot be compared with the rates reported by the report into the 2010 AMS, which are based on all women in the survey and are consequently considerably lower [8]. The all-women total fertility rate reported in the 2010 AMS and based on births in the three years preceding the survey was around 5.0 births per woman. In the 2015 ADHS the reported total fertility rate in the three years preceding the survey was 5.3. This was based on an ever-married sample, but the denominators were inflated before calculating the ASFRs to account for never-married women [6]. It is interesting to observe that the total fertility rate for all women reported for the three years preceding the 2015 ADHS was higher than that reported for the three years preceding the 2010 AMS, consistent with the pattern we find.

Finally, Tables 3 and 4 present the total fertility rate by province and urban or rural residence, along with selected indicators of the proximate determinants of fertility. Table 3 deals with urban areas, and Table 4 deals with rural areas. The first point to note is that, among ever-married women, fertility in Afghanistan is almost the same in urban and rural areas. The lower overall fertility reported from urban areas in the report to the 2015 ADHS, for example, derives from the lower prevalence of marriage in urban areas [6]. The proportion of 20–24 year olds in the 2010 AMS who were married was more than ten percentage points lower in urban areas than in rural areas (Tables 3 and 4).

**Table 3:** Total fertility rates by province: Afghanistan 2008–2010 and 2012–2015, together with measures of proximate determinants of fertility, urban areas.

Region	Province	Total fertility rate		Percentage of 20–24 year old women who are currently married, 2010	Percentage of ever-married women aged 15–49 years who are using modern contraception	Percentage of most recent children born 12–23 months before 2015 AfghanistanDemographic and Health Survey that are still being breastfed
		2008–2010 from Afghanistan Mortality Survey	2012–2015 from Afghanistan Demo-graphic and Health Survey			
Northern	Balkh	7.0	6.5	49.1	25.8	72.9
Northern	Faryab	7.3	8.1	51.4	10.2	70.8
Northern	Jawzjan	5.3	6.0	51.1	17.2	78.2
Northern	Samangan	6.6	6.5	49.1	7.3	80.5
Northern	Sar-E-Pul		6.8		17.2	69.8
North Eastern	Badakhshan	5.7	6.5	60.9	15.9	68.3
North Eastern	Takhar		7.1		16.0	92.2
North Eastern	Baghlan	7.1	6.9	56.3	14.6	76.0
North Eastern	Kunduz	6.4	6.9	58.5	20.5	69.8
Western	Badghis	5.5	6.7	69.2	27.6	75.2
Western	Farah					
Western	Ghor					
Western	Herat		5.6		56.3	77.6
Cen. Highland	Bamyan	na	6.9	na	23.5	78.6
Cen. Highland	Daykundi	na		na		
Capital	Kabul	6.5	6.1	55.0	29.7	69.4
Capital	Kapisa	6.4	6.7	57.1	29.4	62.7
Capital	Logar					
Capital	Panjsher					
Capital	Parwan					
Capital	Wardak					
Southern	Kandahar	7.1	6.8	70.1	38.6	74.7
Southern	Helmand	6.6	6.1	64.4	25.1	64.6
Southern	Nimroz		5.9		31.2	84.7
Southern	Ghazni	6.1	7.2	65.6	21.7	75.2
Southern	Urozgan					
Southern	Zabul					
South Eastern	Khost	5.7	6.9	69.6	16.5	81.9
South Eastern	Paktika		na		na	na
South Eastern	Paktya		6.2		19.0	59.4
Eastern	Kunarha	8.6	8.1	60.1	15.7	78.4
Eastern	Laghman					
Eastern	Nangarhar		7.1		23.8	56.4
Eastern	Nooristan	na	na	na	na	na
Whole country		6.6	6.7	57.9	23.9	71.9

Sources: Afghanistan Mortality Survey 2010 [8]; Afghanistan Demographic and Health Survey 2015 [9].

Notes: Total fertility rates calculated by summing age-specific fertility rates computed using exact exposure of each woman during the periods 2008–2010 and 2012–2015 respectively. The percentages of 12–23 month olds being breastfed are based only on children who survived to the survey date. This table uses unweighted data. na–not available.

**Table 4:** Total fertility rates by province: Afghanistan 2008–2010 and 2012–2015, together with measures of proximate determinants of fertility, rural areas.

Region	Province	Total fertility rate		Percentage of 20–24 year old women who are currently married, 2010	Percentage of ever-married women aged 15–49 years who are using modern contraception	Percentage of most recent children born 12–23 months before 2015 Afghanistan Demographic and Health Survey that are still being breastfed
		2008–2010 from Afghanistan Mortality Survey	2012–2015 from Afghanistan Demo-graphic and Health Survey			
Northern	Balkh	6.3	7.2	54.2	6.0	83.2
Northern	Faryab	6.1	7.2	67.9	9.6	75.5
Northern	Jawzjan	5.9	6.1	60.3	8.6	82.7
Northern	Samangan	6.7	6.4	71.3	4.2	86.0
Northern	Sar-E-Pul	6.4	5.9	70.2	10.2	84.3
North Eastern	Badakhshan	6.1	5.9	76.1	7.4	78.1
North Eastern	Takhar	6.7	7.1	68.8	5.7	73.9
North Eastern	Baghlan	7,5	5.2	72.2	13.9	81.3
North Eastern	Kunduz	4.9	5.7	72.1	9.8	81.7
Western	Badghis	6.2	7.1	76.4	11.9	72.2
Western	Farah	6.3	6.2	68.8	22.1	60.9
Western	Ghor	6.3	6.4	77.9	15.2	90.0
Western	Herat	5.7	6.2	85.0	54.2	62.2
Cen. Highland	Bamyan	7.2	6.6	73.5	21.9	83.3
Cen. Highland	Daykundi	4.6	6.3	89.1	10.4	86.9
Capital	Kabul	8.6	6.8	57.5	22.9	67.9
Capital	Kapisa	6.6	6.7	64.3	16.9	69.4
Capital	Logar	9.2	6.3	64.1	24.5	79.3
Capital	Panjsher	7.4	4.9	58.1	12.5	60.2
Capital	Parwan		7.6		21.6	67.3
Capital	Wardak	7.3	5.9	41.3	29.8	88.2
Southern	Kandahar	na	7.6	na	25.9	73.6
Southern	Helmand	na	5.4	na	13.0	89.2
Southern	Nimroz	4.2	6.1	61.0	26.4	78.8
Southern	Ghazni	5.0	3.5	70.5	12.1	78.6
Southern	Urozgan	9.5	10.0	87.5	10.9	81.9
Southern	Zabul	na	na	na	na	na
South Eastern	Khost	6.2	6.9	70.3	12.2	76.4
South Eastern	Paktika	6.9	6.2	57.4	26.8	52.4
South Eastern	Paktya	6.0	6.4	72.9	9.3	60.4
Eastern	Kunarha	7.4	8.0	64.8	5.0	76.4
Eastern	Laghman	7.7	8.8	77.9	11.9	77.0
Eastern	Nangarhar	6.9	8.6	64.0	9.0	74.3
Eastern	Nooristan	7.3	9.5	53.7	0.8	73.7
Whole country		6.5	6.7	68.6	15.1	71.3

Sources: Afghanistan Mortality Survey 2010 [8]; Afghanistan Demographic and Health Survey 2015 [9].

Notes: Total fertility rates calculated by summing age-specific fertility rates computed using exact exposure of each woman during the periods 2008–2010 and 2012–2015 respectively. The percentages of 12–23 month olds being breastfed are based only on children who survived to the survey date. This table uses unweighted data. na–not available.

A comparison of the reported total fertility rates by province among urban areas suggests either that fertility has increased between 2008–2010 and 2012–2015 in several areas (for example Faryab, Kunduz and Urozgan), or that fertility was under-reported in these areas 2010 AMS relative to the 2015 ADHS. Regional patterns are hard to discern, save perhaps especially high fertility in urban areas in Eastern province. The low fertility in the city of Herat is consistent with a very high (by Afghan standards) prevalence of modern contraception.

In rural areas the figures suggest fertility increases between 2008–2010 and 2012–2015 in several provinces (Balkh, Faryab, Takhar, Kunduz, Badghis, Daykundi, Nimroz, Khost, and all the provinces in Eastern region). However, some of the reported total fertility rates are suspiciously low. In 2008–2010 those in Kunduz, Daykundi and Nimroz provinces seem unreasonably low when compared with the contraceptive prevalence rates among ever-married women, which in Kunduz and Daykundi provinces are also some of the lowest in the country. In 2012–2015 the total fertility rate of 3.5 in Ghazni province seems unreasonably low when the contraceptive prevalence rate of 12.1 per cent is taken into account.

There are also some dramatic changes in the total fertility rate between 2008–2010 and 2012–2015 in provinces such as Logar (a decline from 9.2 to 6.3 in rural areas, but this is based on a sample of fewer than 200 women (Table 1)). In parts of Eastern region, specifically Nangahar and Nooristan provinces, there were increases from 6.9 to 8.6 and from 7.3 to 9.5 respectively, and here the numbers of women are much larger. One possible reason for this is the under-reporting of girl babies in the 2010 AMS. Reported sex ratios at birth in southern Afghanistan in the 2010 AMS were very skewed (125 boys per 100 girls) [8]. The extremely high fertility in Urozgan province is a feature of both surveys, suggesting that it is a real phenomenon. The very high fertility in rural Nooristan province in 2012–2015 is consistent with the near absence of modern contraception.

Breastfeeding for between one and two years is common in all areas of Afghanistan. In all provinces, and in urban and rural areas, at least half the children aged 12–23 months at the time of the 2015 ASHS were being breast fed (Tables 3 and 4). It seems unlikely that geographical variations in fertility are explained to any great extent with geographical differences in breastfeeding behavior.

## Discussion

We focus this discussion eventually on the quality of the birth history data but, first, let us make some remarks about recent fertility trends in Afghanistan. Our results confirm the observations of Thomas Spoorenberg based only on the 2010 AMS that Afghanistan entered its fertility transition around the turn of the century [3]. The fertility transition also looks to be following the classic Asian pattern of an initial decline in fertility among older women, which gradually spreads to include all women aged 25 years and older. There is little evidence of a rural-urban differential in fertility within marriage, though the lower prevalence of marriage in urban areas means that fertility is lower there. There are some regional patterns in fertility, with especially high level being seen in Eastern region and in some other individual provinces, such as Urozgan.

The fertility data in both the 2010 AMS and the 2015 ADHS have their shortcomings. In common with other neighbouring south Asian countries, age heaping on ages ending in the digits 0 and 5 is very pronounced. This has the potential to affect reported fertility rates but the precise effects will depend on the nature of the misreporting (for example whether the tendency is to round ages up to the nearest age ending in the digits 0 or 5, or to round ages down). The age heaping is a feature of both urban and rural populations, but is slightly less prevalent among women with some education than those with no education. Trends in age-specific fertility revealed by the two surveys are broadly consistent, especially at ages 15–19 and 20–24 years. At older ages, the 2015 ADHS reports higher fertility than the 2010 AMS, and there are probably several factors contributing to this, all tending in the same direction.

Regional patterns of fertility are difficult to discern in either survey, and in some provinces there is reason to believe that the birth history data in one or both surveys are deficient. A few provinces have very low reported fertility, and in others there are dramatic increases or decreases in the current and recent fertility levels reported by the 2010 AMS and the 2015 ADHS. Nevertheless, some regional patterns seem robust. High fertility seems characteristic of Eastern region and rural areas of Urozgan province. The city of Herat has low fertility and a high contraceptive prevalence rate.

## Conclusion

Afghanistan has only conducted two nationally representative surveys which collected birth history data: the 2010 Afghanistan Mortality Survey (AMS) and the 2015 Afghanistan Demographic and Health Survey (ADHS). In this paper we have examined the accuracy of age reporting in the two surveys, the consistency between the estimates of the trends in age-specific fertility they generate, and the plausibility of the provincial fertility estimates when set alongside estimates of the proximate determinants of fertility.

Our conclusion is that both surveys have weaknesses, notably in the reporting of women’s ages. The 2010 AMS suffers from the omission of female births in the south of the country, and perhaps among older women, but this was rectified to some extent in the 2015 ADHS. Some provinces have unusually low fertility rates given the reported rates of use of modern contraception. Despite this, the two surveys both reveal the onset of the fertility transition in Afghanistan around the turn of the century, and there is a broad consistency in the reported trends in age-specific fertility. Overall, we conclude that the fertility data in the two surveys can be used with care to give an indication of broad regional fertility patterns and trends in the country.
